# Supplementary material for: Transforming students’ attitudes towards learning through the use of successful educational actions
Source: PLoS One. 2020 Oct 12;15(10):e0240292. doi: 10.1371/journal.pone.0240292 (PMC7549835; doi:10.1371/journal.pone.0240292)
Supplement: S1 File — (PDF) [file pone.0240292.s001.pdf]

What I think about learning in school

Meet Sam ….. 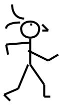 …..

Sam goes to school like you and is interested in what other children think about learning in school. We would like to know what **you** think about learning, to make schools better for children in the future. Here are some other children's views about learning and would like to know whether you agree or disagree with them.

Please give your feeling about every one, as quickly as you can, by making a tick mark in the box you choose. There are no right and wrong answers. Just make sure you **tick what you really feel.** Your answers are important. Don't show the tick marks to anyone else.

Your Name* ……………….……………………   Your age ……………

Your School ……………………………..…………   Your Class …………………….

What I really think about learning in school

Do you strongly agree, agree a little, disagree a little, strongly disagree , or are you not sure ?

Please put a tick in the box you choose.

	View	Stongly agree	Agree a little	Not sure	Disagree a little	Strongly disagree
**1**	We learn best when the teacher tells us what to do.					
**2**	We can learn more when we can express our own ideas.					
**3**	Learning through discussion in class is confusing.					
**4**	Sometimes, learning in school is boring.					

	View	Stongly agree	Agree a little	Not sure	Disagree a little	Strongly disagree
**5**	Learning in school is better when we have other adults to work with us.					

**6**	I would rather think for myself than hear other people's ideas.					
**7**	I enjoy learning when my friends help me					
**8**	It is good to hear other people's ideas.					
**9**	Helping my friends has helped me to understand things better.					
**10**	I am more confident about learning in school than I used to be.					
**11**	I like discussing the books we read with the class.					
**12**	At home, sometimes we talk about what I have been learning in school.					

The spaces below are for you to write what you think about learning in DLG or Interactive Groups .
Please circle the one you are writing about.

We are doing **Interactive groups** **DLG**

It is **good** because ...

A **not so good** thing about it is …<br>because ...

Did you answer all the questions? You did? Thank you very much ... 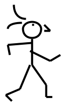 …

- * *We will not use your name or show your paper to other people, but we need your name for now so that we don't mix up your answers with other people's answers.*

© *Dr Anthony Pell* The Sam Attitudes Questionnaires are the intellectual property of Dr Anthony Pell
