# Supplementary material for: Transforming students’ attitudes towards learning through the use of successful educational actions
Source: PLoS One. 2020 Oct 12;15(10):e0240292. doi: 10.1371/journal.pone.0240292 (PMC7549835; doi:10.1371/journal.pone.0240292)
Supplement: Attachment — Submitted filename: rebuttal letter_2.docx [file pone.0240292.s003.docx]

Barcelona, September 16^th^, 2020

Dear Anita Estes,

Regarding your last email on the article “Transforming Students’ Attitudes Towards Learning Through the Use of Successful Educational Actions”, we have attaching here the new version of that article.

In your email, you requested:

1) Please ensure that you refer to Table 14 in your text as, if accepted, production will need this reference to link the reader to the Table.
**Table 14 is referred in page 23, line 434; it is placed in page 24, line 450.**
2) Please remove your figures/ from within your manuscript file, leaving only the individual TIFF/EPS image files.  These will be automatically included in the reviewer’s PDF

**Figures 1 and 2 have been removed according to this comment and attached as tiff archives. In the manuscript file, they have been referred as [S1 Fig 1] and [S2 Fig 2] in lines 382-84 and 415-16 respectively.**

We hope that now the manuscript is ready for your consideration.

Sincerely,

Javier Díez-Palomar, Rocío García-Carrión, Linda Hargreaves and María Vieites
